# Comparative Evaluation of the Anesthetic Efficacy of Needle-Free Anesthesia and Conventional Anesthesia in Patients With Symptomatic Irreversible Pulpitis: A Randomized Clinical Trial

**DOI:** 10.7759/cureus.54661

**Published:** 2024-02-21

**Authors:** Krishna Kanth Jaju, Iffat Nasim, Sahil Choudhari, Hima Sandeep A

**Affiliations:** 1 Conservative Dentistry and Endodontics, Saveetha Dental College and Hospitals, Saveetha Institute of Medical and Technical Sciences (SIMATS) Saveetha University, Chennai, IND

**Keywords:** needle-free anesthesia, pain, local anesthesia, irreversible pulpitis, conventional syringe

## Abstract

Introduction

Pain is the primary reason for which most of the patients seek endodontic treatment. Local anesthesia is considered to be the most important step in the procedure to reduce the pain. However, the majority of the patients do not cooperate due to the fear of syringe anesthesia. The aim of this clinical trial was to compare the anesthetic efficacy of needle-free anesthesia and conventional anesthesia in patients with symptomatic irreversible pulpitis undergoing root canal therapy.

Materials and methods

A total of 54 patients were enrolled in the study, and the treatment was administered by a single operator. The initial assessment of vitality included cold testing, heat testing, and electric pulp testing. Preoperative pain was assessed using the Visual Analog Scale (VAS) before the administration of anesthesia. Local anesthesia was administered according to the group assigned: Group 1 (conventional anesthesia) and Group 2 (needle-free anesthesia). The pain was assessed during the administration of anesthesia. Following the administration of anesthesia, the vitality of the tooth was evaluated using cold testing, heat testing, and electric pulp testing. Subsequently, the tooth was isolated with a rubber dam, and the access cavity was prepared. The pain was assessed during access cavity preparation and during the first file insertion. Working length was determined using an apex locator (Root ZX Mini, J Morita, Saitama, Japan) and was confirmed using intraoral periapical radiographs. Later on, further treatment was carried out.

Results

A total of 54 participants were included in this clinical trial. There was no significant difference in mean age distribution between the two groups (p=0.852). Considering the frequency distribution of gender, there was no significant difference; however, Group 1 had more female participants (59.3%) compared to Group 2 (33.3%). There was a significant reduction in the mean pain score in Group 2 compared to Group 1 during the delivery of anesthetic agents (p=0.000).

Conclusion

Needle-free anesthesia proves to be equally effective as the conventional syringe system in patients experiencing symptomatic irreversible pulpitis undergoing root canal treatment. However, it is noteworthy that patients exhibited greater comfort levels with needle-free anesthesia systems specifically during the administration of the anesthetic solution.

## Introduction

Pain is the primary reason for which most of the patients seek endodontic treatment [1]. Local anesthesia is a pivotal component of pain management in dental procedures, and the quest for improved techniques remains a focal point in contemporary dentistry [2]. Symptomatic irreversible pulpitis, characterized by severe and persistent toothache, demands meticulous attention to pain control during endodontic interventions [3]. The majority of the population do not visit dentists regularly mostly due to the fear of the needle which could be quite distressing for the patients, and also a negative impact can be created on the patient's psychology as the needle penetrates the oral mucosa [4]. Needle-free anesthesia, utilizing methods such as jet injection or transmucosal drug delivery, has emerged as a novel alternative to traditional needle-based techniques [5,6]. Proponents argue that it not only eliminates the anxiety associated with needle phobia but also provides a more controlled and precise administration of local anesthetics [7,8]. It is essential to discern whether this innovative approach can offer comparable or superior anesthetic outcomes compared to conventional methods, thereby potentially revolutionizing pain management strategies in symptomatic irreversible pulpitis.

The decision to focus on symptomatic irreversible pulpitis is rooted in the clinical significance of this condition and the associated challenges in achieving profound anesthesia. Patients with irreversible pulpitis often present with heightened pain sensitivity, making it an ideal cohort to assess the nuances of anesthetic efficacy [9]. The aim of this randomized clinical trial was to compare the anesthetic efficacy using the Visual Analog Scale (VAS) of needle-free anesthesia and conventional anesthesia in patients with symptomatic irreversible pulpitis.

## Materials and methods

The clinical trial received approval from the Institutional Review Board at Saveetha Institute of Medical and Technical Sciences in Chennai, India (approval number: SRB/SD/MDS03/18/ODS/10), and was registered with "Clinical Trials Registry of India" (reference number: CTRI/2021/12/038471). The research design employed a prospective, single-centered, parallel randomized double-blinded controlled trial. The clinical trial followed the protocol established by the Consolidated Standards of Reporting Trials (CONSORT) statement. 

Included were patients between 18 and 40 years of age and with periodontally healthy teeth, teeth with symptomatic irreversible pulpitis with periapical index (PAI) score ≤3, and restorable teeth, while patients with pulpal necrosis, systemic diseases, allergies to local anesthetics, and teeth with periapical lesions were excluded. Volunteer patients meeting the aforementioned inclusion criteria were enrolled in the investigation. Participants were sourced from eligible individuals within the Department of Conservative Dentistry and Endodontics at Saveetha Dental College and Hospitals in Chennai, India.

**Figure 1:**
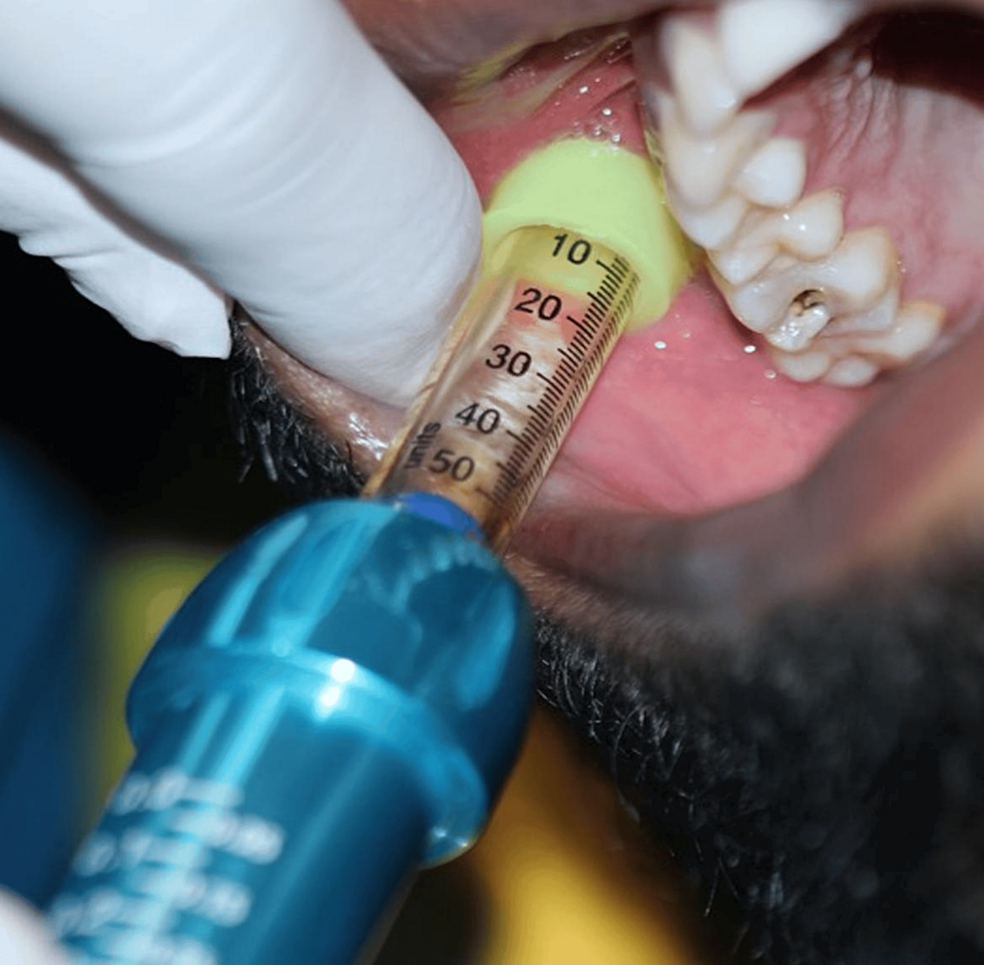
Needle-free system

The randomization process was conducted in advance by a third party with no connection to the study. Employing a block randomization procedure, participants were allocated to either Group 1 (conventional anesthesia (n=27)) or Group 2 (needle-free anesthesia (n=27)). Jet injector was used as the needle-free anesthesia in the current study (Figure 1). The allocation concealment utilized the SNOSE (sequentially numbered, opaque, sealed envelopes) method, ensuring the sequence remained undisclosed until interventions were assigned. A third party prepared opaque, sealed envelopes, each containing a randomized group number and marked with a serial number. Participants were sequentially assigned study numbers upon entry into the study. The envelope was opened only when the intervention was to be assigned, revealing the designated group for the respective participant. Treatment was administered based on the group indicated in the enclosed paper. To maintain blinding, both patients and the evaluator were kept unaware of the treatment type and protocol. However, the operator was informed about the treatment type and protocol, resulting in the implementation of double blinding.

Prior to commencing treatment, a comprehensive medical and dental history was meticulously collected. The preoperative information for each patient, encompassing age, sex, and tooth number, was documented in a prearranged patient chart. Qualifying patients were apprised of the treatment and study design, and voluntary participation was sought, with informed consent obtained from those willing to join the study. The 54 patients who provided informed consent were randomly assigned to two groups: Group 1 (conventional anesthesia) and Group 2 (needle-free anesthesia). The preoperative pain severity was assessed using VAS, with pain levels recorded numerically and verbally on a scale of 0-10, denoting no pain (0), mild pain (1-3), moderate pain (4-6), and severe pain (7-10).

A single operator conducted the treatment, beginning with the assessment of vitality through cold, heat, and electric pulp testing. Preoperative pain levels were evaluated using VAS before the administration of anesthesia. Local anesthesia was administered according to the group assigned. Lidocaine HCl 2% with epinephrine 1:100,000 was used as a local anesthetic solution. The pain was assessed during the administration of anesthesia using VAS. After the administration of anesthesia, the vitality of the tooth was assessed using cold testing, heat testing, and electric pulp testing. The tooth was isolated using a rubber dam, and the access cavity was prepared using Endo access bur size 2 (Dentsply Sirona, Charlotte, NC) and sterile carbide burs. The pain was assessed during access cavity preparation and during the first file insertion using VAS. Working length was determined using an apex locator (Root ZX Mini, J Morita, Saitama, Japan) and was confirmed using intraoral periapical radiographs. Later on, further treatment was carried out.

Statistical analysis

Sample size determination was conducted a priori using G*Power 3.1.2 software (Heinrich Heine University Düsseldorf, Germany) [10]. The minimum sample size for each group was computed under the conditions of a power of 0.90 and p≤0.05, resulting in a total sample size of 54 (27 participants per group). Statistical analysis was carried out using IBM SPSS Statistics for Windows, V. 21.0 (IBM Corp., Armonk, NY). Normality tests, including the Kolmogorov-Smirnov and Shapiro-Wilk, indicated that all variables followed a normal distribution. Consequently, parametric tests were employed for data analysis. A p-value of <0.05 was considered statistically significant for the tests conducted. The Mann-Whitney U test was utilized to compare pain scores and pain during the delivery of the anesthetic solution between groups. An independent t-test was performed to evaluate the mean difference between treatment groups. Additionally, a two-way ANOVA test was conducted to assess significant differences in pain scores within the treatment groups.

## Results

**Figure 2:**
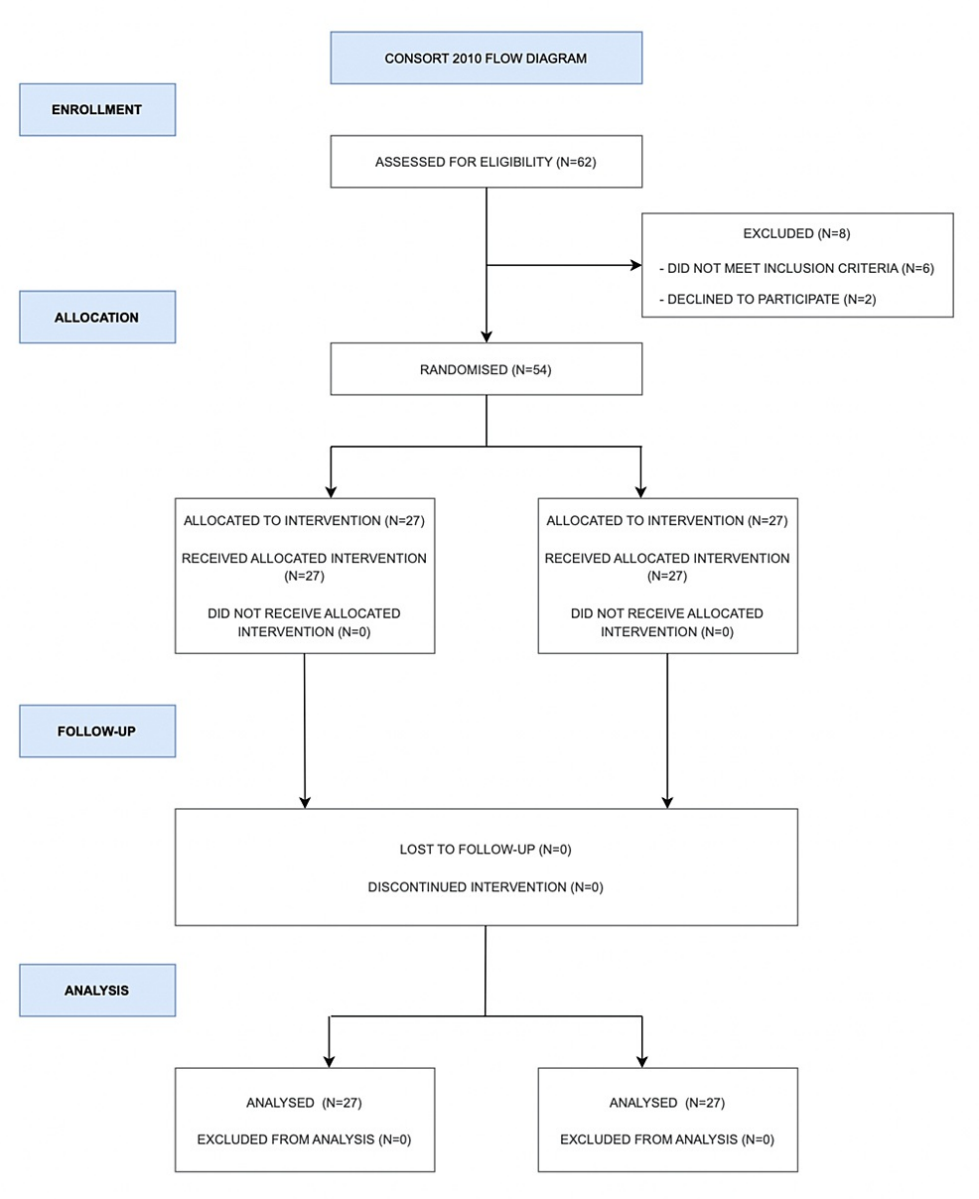
CONSORT 2010 flow diagram CONSORT: Consolidated Standards of Reporting Trials

In the present clinical trial, 62 patients were assessed for eligibility. Eight patients did not meet the inclusion criteria and were excluded from the trial. The clinical trial comprised 54 participants. No patients were excluded from the analysis (Figure 2). 

All patients tested positive on evaluation using heat testing, cold testing, and electric pulp testing. An analysis of the age distribution revealed no statistically significant difference between Group 1 and Group 2 (p=0.852), suggesting a comparable age profile within the two study groups. This observation suggests that any observed effects on the primary outcome variable are less likely to be attributed to age-related factors. Regarding gender distribution, although no statistically significant distinction was found, it is noteworthy that Group 1 exhibited a higher proportion of female participants (n=16, 59.3%) compared to Group 2 (n=9, 33.3%). While not a primary focus of the study, this gender imbalance may be considered in the interpretation of results, and further investigation could be warranted to explore potential gender-related influences. 

There was no statistical difference in the pre-op pain score, access opening, and file insertion between the two groups (p>0.05). The primary objective of this study centered around the administration of anesthetic agents. During this process, Group 2 displayed a substantial reduction in mean pain scores in comparison to Group 1, with a highly significant p-value of 0.000. This compelling finding strongly supports the hypothesis that the anesthetic approach employed in Group 2 resulted in a notable decrease in pain experienced by participants (Table 1).

**Table 1:** Distribution of pain score (VAS score) There was no significant reduction in the mean pre-op pain score and pain during access opening and file insertion. There was a significant reduction in the mean pain score in Group 2 compared to Group 1 during the delivery of the anesthetic agent (p=0.000). p<0.05 is considered as statistically significant VAS: Visual Analog Scale; SD: standard deviation

Pain	Mean±SD (VAS score)		P-value
	Group 1	Group 2	
Pre-op	7.63±0.63	7.63±0.63	0.813
Access opening	0.00±0.00	0.00±0.00	1.000
File insertion	0.04±0.19	0.00±0.00	0.317
During the delivery of the anesthetic agent	5.74±1.06	0.00±0.00	0.000

## Discussion

The results of the present study showed similar efficacy of needle-free anesthesia technique and conventional anesthesia technique in patients with symptomatic irreversible pulpitis and none of the patients required additional anesthesia. Lidocaine HCl 2% with epinephrine 1:100,000 was used as a local anesthetic solution.

Dabarakis et al. [11] and Makade et al. [12] compared both needle and pressure anesthesia and concluded both techniques had similar efficacy. In both studies, a similar anesthetic solution was used. In a trial conducted by Christensen et al., 60% of the patients required additional anesthesia following the use of the Numbee Needle-less Dental Anesthesia Delivery System [13]. In another study, traditional infiltration was more effective compared with the needleless INJEX (Rösch, Berlin, Germany). Around 80.5% of the patients required additional anesthesia following the use of INJEX, a needle-free injection system [14]. Ocak et al. [15] reported there was a need for additional anesthesia during extraction after jet injection. The authors used 0.3 ml of articaine 4% as a local anesthetic solution. However, Saleh et al. [16], using a higher dose of the same anesthetic solution (0.6 ml of articaine 4%), found no significant difference between the needle-free anesthesia technique and conventional anesthesia technique.

In the present study, on assessing pain felt during the administration of anesthesia, there was a significant difference between the groups (p<0.05). Out of 27 patients, none of the patients felt pain at the site of injection during the administration of anesthesia with the Comfort-in jet injector (Mika Medical Co, Busan, Korea), but they experienced ​​discomfort and fear. This may be due to the sudden pressure as well as the noise produced during the discharge of the solution. During the administration of anesthesia with a conventional syringe, all 27 patients felt pain; this might be due to the sharp needle piercing the oral mucosa. Makade et al. [12] also demonstrated higher discomfort but significantly less fear with jet injection as compared to conventional technique in adult patients. Oliveira et al. [17] concluded that both the needle and pressure anesthesia techniques did not differ concerning the pain experienced during anesthesia. Ocak et al. [15] demonstrated that patients experienced more discomfort when the needle was in contact with the tissues compared to the jet device. Arapostathis et al. [14] found that more than 50% of the participants experienced pain or discomfort and fear during the administration of anesthesia using jet injection in children of age group 6-11 years.

In the present study, none of the patients reported pain during access opening after the administration of local anesthesia using both techniques. None of the patients felt pain during the first file insertion using the needleless anesthesia technique; some patients felt pain during the first file insertion after the administration of local anesthesia using the conventional anesthesia technique, although the results were not statistically significant (p>0.05). None of the patients reported postoperative pain, ecchymosis, or lacerations in both techniques after anesthesia.

The current trial utilizes a needleless anesthesia device, but it comes with certain limitations. The device features a straight angulation, a smaller nozzle diameter, and a larger overall size. To address these drawbacks, future iterations should consider a smaller device size and a larger nozzle diameter. This improvement aims to minimize additional anesthesia requirements, alleviate patient fears, foster a positive attitude, and enhance patient cooperation during dental treatments. Conducting further studies with a substantial sample size across a range of dental procedures, both invasive and non-invasive, would greatly contribute to obtaining more comprehensive and valuable results.

## Conclusions

Within the limitations of the present study, it can be concluded that needle-free anesthesia was as efficient as the conventional syringe system in patients with symptomatic irreversible pulpitis undergoing root canal treatment. However, patients were more comfortable with needle-free anesthesia systems during the injection of the anesthetic solution.

## References

[1] Rechenberg DK, Held U, Burgstaller JM, Bosch G, Attin T (2016). Pain levels and typical symptoms of acute endodontic infections: a prospective, observational study. BMC Oral Health.

[2] St George G, Morgan A, Meechan J, Moles DR, Needleman I, Ng YL, Petrie A (2018). Injectable local anaesthetic agents for dental anaesthesia. Cochrane Database Syst Rev.

[3] Drum M, Reader A, Nusstein J, Fowler S (2017). Successful pulpal anesthesia for symptomatic irreversible pulpitis. J Am Dent Assoc.

[4] McLenon J, Rogers MA (2019). The fear of needles: a systematic review and meta-analysis. J Adv Nurs.

[5] Gao Q, Noël G, Der Khatchadourian Z (2021). Needle-free injection: dental infiltration anesthesia. Int J Pharm.

[6] Belevcikli M, Altan H, Demir O (2023). Effect of the new needle-free injection system on pain perception and dental anxiety during anesthesia: randomized controlled split-mouth study. J Dent Anesth Pain Med.

[7] Yıldırım S, Tokuç M, Aydın MN (2020). The effect of pre-anesthesia with a needle-free system versus topical anesthesia on injection pain of the inferior alveolar nerve block: a randomized clinical trial. Clin Oral Investig.

[8] Brunton PA, McLean M, Vedagiri S (2022). Jet injection needle-free dental anaesthesia: initial findings. J Dent.

[9] Costa YM, de Souza PR, Marques VA, Conti PC, Vivan RR, Duarte MA, Bonjardim LR (2020). Intraoral somatosensory alterations impact pulp sensibility testing in patients with symptomatic irreversible pulpitis. J Endod.

[10] Kang H (2021). Sample size determination and power analysis using the G*Power software. J Educ Eval Health Prof.

[11] Dabarakis NN, Alexander V, Tsirlis AT, Parissis NA, Nikolaos M (2007). Needle-less local anesthesia: clinical evaluation of the effectiveness of the jet anesthesia Injex in local anesthesia in dentistry. Quintessence Int.

[12] Makade CS, Shenoi PR, Gunwal MK (2014). Comparison of acceptance, preference and efficacy between pressure anesthesia and classical needle infiltration anesthesia for dental restorative procedures in adult patients. J Conserv Dent.

[13] Christensen C, Arnason SC, Oates R, Crabtree M, Kersey JW, Vandewalle KS (2020). Efficacy of pulpal anesthesia using a needle-less syringe. Anesth Prog.

[14] Arapostathis KN, Dabarakis NN, Coolidge T, Tsirlis A, Kotsanos N (2010). Comparison of acceptance, preference, and efficacy between jet injection INJEX and local infiltration anesthesia in 6 to 11 year old dental patients. Anesth Prog.

[15] Ocak H, Akkoyun EF, Çolpak HA, Demetoğlu U, Yücesoy T, Kılıç E, Alkan A (2020). Is the jet injection effective for teeth extraction?. J Stomatol Oral Maxillofac Surg.

[16] Saleh G, Michaelis A, Lang H, Raab W (2005). Anesthetic effective potential of a needle-free injection system. Quintessenz.

[17] Oliveira AC, Amorim KS, Nascimento Júnior EM, Duarte AC, Groppo FC, Takeshita WM, Souza LM (2019). Assessment of anesthetic properties and pain during needleless jet injection anesthesia: a randomized clinical trial. J Appl Oral Sci.

